# The Association of Normal Range Glycated Hemoglobin with Restrictive Lung Pattern in the General Population

**DOI:** 10.1371/journal.pone.0117725

**Published:** 2015-02-06

**Authors:** Il Hwan Oh, Jung Hwan Park, Chang Hwa Lee, Joon-Sung Park

**Affiliations:** Department of Internal Medicine, College of Medicine, Hanyang University, Seoul, Korea; Azienda Ospedaliero-Universitaria Careggi, ITALY

## Abstract

Glycated hemoglobin (HbA1c) is an important diagnostic indicator of diabetes mellitus, and some authors have argued that it is related to impaired lung function in the diabetic population. However, there was rare study for association between lung function and HbA1c in the non-diabetic population. We investigated whether HbA1c below the diagnostic threshold is related to deficits in lung function. We analyzed biochemical and spirometry data from a nation-wide, population-based, case-control study (the KNHANES IV and V). Eligible as cases were all native Koreans aged 40 years or more with no medical illness. A total of 3670 participants were divided into 4 groups according to HbA1c (%) as follows: Group I (n = 842), ≥ 4.0 and ≤ 5.3; Group II (n = 833), > 5.3 and ≤ 5.5; Group III (n = 898), > 5.5 and ≤ 5.7; and Group IV (n = 1097), > 5.7 and ≤ 6.4. Group I had the greatest forced vital capacity (FVC, 96.3 ± 0.5% *pred*, P < 0.0001), forced expiratory volume per second (FEV_1_, 93.8 ± 0.5% *pred*, P < 0.0001) and FEV_1_/FVC (0.792 ± 0.003, P < 0.0001) compared with the other groups. Linear regression showed that HbA1c was closely related to FVC (β = -6.972154, P < 0.0001) and FEV_1_ (β = -5.591589, P < 0.0001), but not to FEV_1_/FVC. Logistic regression analysis revealed a significant association between HbA1c and a restrictive spirometric pattern (FVC < 80% *pred.*, FEV_1_/FVC ≥ 0.70; OR = 3.772, 95% CI = 1.234-11.53), indicating that elevated HbA1c is closely associated with lung impairment in the non-diabetic population. In the healthy population, relatively high HbA1c level is associated with decrements of FVC and FEV_1_ and may be a reliable predictor of poor lung function, especially the restrictive pattern.

## Introduction

Diabetes mellitus (DM) has become a global pandemic due to population growth, aging, urbanization, and the increasing prevalence of obesity and physical inactivity [1], and it accounts for the majority of the social and economic burden among patients and society in general [2].

Dysglycemia is one of the major factors responsible for the development of micro- and macro-angiopathies via several potential molecular mechanisms [3]. Dysglycemia over-production of reactive oxygen species (ROS) and changes in various signaling pathways have been reported to cause multiple types of vascular dysfunction and inhibit endogenous vascular protective mechanisms [3]. Thus, dysglycemia may induce vascular complications in all organs with large vascular network systems.

The lung is also an organ susceptible to diabetic microvascular complications [4]. Previous studies have shown that microangiopathic processes and biochemical changes of elastic recoil properties are related to the development of lung complications in the diabetic population [4]. Alterations of collagen and elastin fibers and thickening of alveolar capillaries and pulmonary arteriolar walls may result in thickening of the alveolar epithelial basal lamina, leading to reduced pulmonary capacity for gas exchange [5].

Many studies have shown that hyperglycemia is related to poor lung function in patients with full-grown DM [5–8]. Interestingly, some authors have argued that the initiation and development of vascular complications are not limited to diabetic patients and that, even in the prediabetic state, various DM-associated risk factors may contribute to micro- and macro-angiopathy as part of diabetic complications [9]. However it is not clear how serum glucose levels in the prediabetic or non-diabetic states begin to affect lung function.

Glycated hemoglobin (HbA1c), a glycated form of hemoglobin A, reflects the mean blood glucose level over the preceding 2–3 months, and is frequently measured for diagnosing DM and monitoring glycemic control [10]. Epidemiological studies have demonstrated that a rise in HbA1c increases the risk of cardiovascular disease in diabetic and non-diabetic populations [11, 12]. Furthermore, Pinto Pereira et al. have demonstrated an association between HbA1c and reduced lung function in diabetic individuals [6]. However, little is known about the relation between HbA1c level and poor lung function, especially in generally healthy individuals. In this study, we investigated whether HbA1c level is related to impaired lung function in the general population.

## Materials and Methods

### Study population

The data were collected from publicly available data sets of the Korean National Health and Nutrition Examination Survey (KNHANES) conducted by the Korea Centers for Disease Control and Prevention (KCDC) among non-institutionalized Korean civilians between 2008 and 2012. All the participants volunteered and provided written informed consent prior to their enrollment. All participants’ records, apart from survey date and home region, were anonymized prior to analysis, and the study was approved by the Institutional Review Board (IRB) of the Korea Centers for Disease Control and Prevention (IRB: 2008-04EXP-01-C, 2009-01CON-03-2C, 2010-02CON-21-C, 2011-02CON-06-C, 2012-01EXP-01-2C).

**Fig 1:**
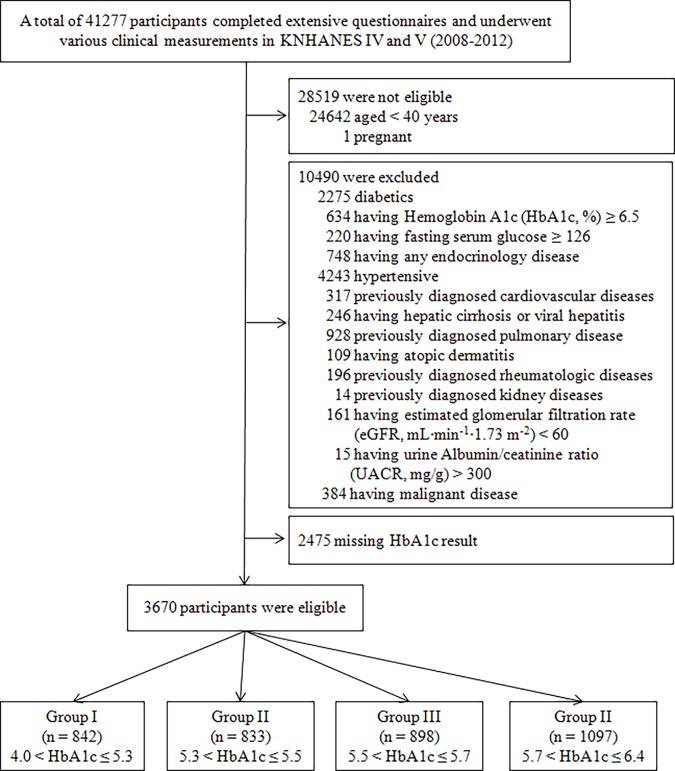
Flow chart of the study group enrollment process. KNHANES, Korean National Health and Nutritional Examination Survey.

A total of 41277 individuals participated in the KHANES 2008–2012. The following were excluded from this study: participants for whom data were lacking (anthropometric or laboratory data), those under 40 years of age, those with any medical problems or chronic kidney disease with estimated glomerular filtration rate (eGFR, mL·min^-1^·1.73 m^-2^) below 60. The total number of eligible participants was 3670 (Fig. 1).

### Anthropometric and Clinical Measurements

Anthropometric measurements were made by well-trained examiners. Height was measured to the nearest 0.1 cm using a portable stadiometer (Seriter, Bismarck, ND). Waist circumference (WC) was measured using flexible tape at the narrowest point between the lowest border of the rib cage and the uppermost lateral border of the iliac crest at the end of normal expiration. Waist/height ratio (WHtR) was calculated as the ratio of WC (cm) and height (cm). The conicity index (C-index) was computed, via a mathematical expression, using the measurement of weight, height, and WC [13].

Blood pressure (BP) was measured three times with a mercury sphygmomanometer (Baumanometer; Baum, Copiague, NY), in a seated position after at least 5 min rest. The average values of the three recorded systolic and diastolic BPs were used in the analyses.

### Laboratory Methods

Venous blood was sampled after an 8 h overnight fast. Fasting plasma concentrations of glucose and lipids were measured enzymatically in a central laboratory using a Hitachi Automatic Analyzer 7600 (Hitachi, Tokyo, Japan). HbA1c levels were determined by high performance liquid chromatography using an automated HLC-723G7 analyzer (Tosoh Corporation, Tokyo, Japan). Serum concentrations of **creatinine** were measured by a colorimetric method (Hitachi **Automatic Analyzer 7600**), and eGFR was calculated using the Chronic Kidney Disease Epidemiology Collaboration (CKD-EPI) equation [14].

### Pulmonary Function Testing and Poor Lung Function Patterns

Spirometry tests to determine lung function patterns were performed in participants over 40 by trained technicians using dry rolling-seal spirometers (Sensor Medics, Yorba Linda, USA) [15]. The technicians were trained to review the spirometry test results according to the American Thoracic Society/European Respiratory Society criteria. The spirometry data had to fulfill two criteria: (1) two or more acceptable spirometry curves had to be generated to ensure correct inspiration and 6-s expiration measurements and (2) there had to be a maximum of 50-mL inter-measurement variability in forced vital capacity (FVC) and forced expiratory volume per second (FEV_1_). The spirometry tests were undertaken without a bronchodilator. Age-, sex- and height-adjusted normal predicted values for FVC and FEV_1_ in the Korean general population were used to calculate the values of % predicted FVC and FEV_1_ [16]. The restrictive pattern was defined as a percent predicted value of FVC < 80% and FEV_1_/FVC ≥ 0.7, and the obstructive pattern as an FEV_1_/FVC ratio < 0.7 [15].

### Statistical Analysis

Data are presented as means ± SE, or frequencies (and proportions). The *t*-test was employed to compare quantitative variables and Pearson’s chi-square test to compare proportions for categorical variables. The data were analyzed with sampling weights to account for multistage and stratified sampling. Linear regression analysis was used to identify factor(s) related to spirometric parameters in the study participants. Odds ratios (ORs) with 95% confidence intervals (95% *CI*s) were calculated in multiple logistic regression models according to poor lung function (normal vs. restrictive). Two-tailed P < 0.05 was considered statistically significant. All statistical analyses were performed with Statistical Analysis Software (version 9.2; SAS Institute Inc, Cary, NC, USA).

## Results

### Baseline and spirometric characteristics

Participants consisted of 1580 men and 2090 women with a mean age of 54.8 ± 11.6 years. Their anthropometric, clinical, laboratory, and spirometric characteristics of the study population according to their HbA1c levels are listed in Table 1. Group IV were older and smoked more cigarettes than the other groups. Participants with higher HbA1c levels suffered more from hypertension, were more obese, and also had worse lipid profiles and poorer kidney function than those with lower HbA1c levels in the non-diabetic population. FVC (92.9 ± 0.4% *pred*, P < 0.0001), FEV_1_ (91.5 ± 0.5% *pred*, P = 0.0006), and FEV_1_/FVC (0.777 ± 0.003, P = 0.0002) were significantly lower in Group IV than in the other groups. In addition, the highest HbA1c group had the highest proportion of individuals with obstructive and restrictive patterns.

**Table 1:** Baseline and spirometric characteristics of the 3670 participants.

Parameter	Group I	Group II	Group III	Group IV	P
	4.0 ≤HbA1c ≤ 5.3	5.3 < HbA1c ≤ 5.5	5.5 < HbA1c ≤ 5.7	5.7 < HbA1c ≤ 6.4	
	(n = 842)	(n = 833)	(n = 898)	(n = 1097)	
Age (year)	44.8 ± 0.3	50.4 ± 0.4	52.2 ± 0.4	54.5 ± 0.4	<0.0001
Male gender (%)	363 (43)	363 (44)	391 (44)	463 (42)	0.7485
Systolic blood pressure (mm Hg)	117.3 ± 0.7	118.0 ± 0.6	117.9 ± 0.7	120.6 ± 0.7	0.0005
Diastolic blood pressure (mm Hg)	78.1 ± 0.5	77.8 ± 0.5	77.3 ± 0.4	78.2 ± 0.4	0.9780
Body mass index (kg/m^2^)	23.2 ± 0.1	23.6 ± 0.1	23.6 ± 0.1	24.4 ± 0.1	<0.0001
Waist circumference (cm)	79.3 ± 0.4	80.7 ± 0.4	81.0 ± 0.4	84.0 ± 0.3	<0.0001
Waist/height ratio	0.486 ± 0.002	0.496 ± 0.002	0.499 ± 0.002	0.519 ± 0.002	<0.0001
Conicity index (m^1½^·kg^-½^)	1.183 ± 0.003	1.195 ± 0.003	1.200 ± 0.003	1.226 ± 0.003	<0.0001
Hemoglobin (mg/dL)	14.3 ± 0.1	14.3 ± 0.1	14.1 ± 0.1	14.1 ± 0.1	0.0245
Ferritin (ng/dL)	96.0 ± 8.3	87.4 ± 4.1	83.3 ± 4.1	93.4 ± 4.1	0.7087
Creatinine (mg/dL)	0.83 ± 0.01	0.84 ± 0.01	0.84 ± 0.01	0.84 ± 0.01	0.3448
eGFR*(mL·min^-1^·1.73 m^-2^)	95.1 ± 0.5	93.9 ± 0.5	92.1± 0.5	91.2 ± 0.5	<0.0001
Glucose (mg/dL)	90.4 ± 0.3	92.2 ± 0.4	93.5 ± 0.4	98.6 ± 0.4	<0.0001
HbA1c (%)	5.146 ± 0.008	5.455 ± 0.002	5.647 ± 0.002	5.969 ± 0.006	<0.0001
Triglyceride (mg/dL)	102.4 ± 3.4	135.8 ± 5.3	137.7 ± 3.3	158.5 ± 5.1	<0.0001
HDL-cholesterol (mg/dL)	55.0 ± 0.0	48.4 ± 4.6	56.4 ± 1.9	60.9 ± 0.0	0.5508
LDL-cholesterol (mg/dL)	113.8 ± 3.0	119.1 ± 3.0	122.5 ± 2.5	131.7 ± 2.6	<0.0001
25-Vitamin D (ng/dL)	17.5 ± 0.3	17.6 ± 0.3	17.7 ± 0.2	18.1 ± 0.3	<0.0001
Current smoker (%)	140(17)	161 (19)	175 (19)	215(20)	0.6184
Ex-smoker (%)	117 (21)	150 (18)	168 (19)	196 (18)	0.8729
Smoking amount (peak·year)^†^	7.7 ± 0.6	8.8 ± 0.6	9.3 ± 0.6	10.3 ± 0.6	0.0036
FVC (%, *predicted*)	96.3 ± 0.5	96.2 ± 0.5	95.1 ± 0.5	92.9 ± 0.4	<0.0001
FEV1 (%, *predicted*)	93.8 ± 0.5	93.8 ± 0.5	93.2 ± 0.6	91.5 ± 0.5	0.0006
FEV_1_/FVC	0.792 ± 0.003	0.786 ± 0.003	0.784 ± 0.003	0.777 ± 0.003	0.0002
FEF_25–75_ (L/sec)	3.02 ± 0.04	3.00 ± 0.05	2.92 ± 0.05	2.73 ± 0.04	<0.0001
PEF (L/sec)	7.71 ± 0.08	7.83 ± 0.09	7.65 ± 0.09	7.42 ± 0.08	0.0064
Spirometric abnormality					
Obstructive (%)	53 (6)	66 (8)	72 (8)	144 (10)	0.0031
Restrictive (%)	34 (4)	35 (4)	42 (5)	90 (8)	0.0151

Results are expressed as mean ± SE or frequencies (and proportions).

HbA1c, hemoglobin A1c; eGFR, estimated glomerular filtration rate; HDL, high-density lipoprotein; LDL, low-density lipoprotein; FVC, forced vital capacity; FEV_1_, forced expiratory volume in one second; FEF_25–75_, forced expiratory flow, mid-expiratory phase; PEF, peak expiratory flow.

*Kidney function was estimated using the Chronic Kidney Disease Epidemiology Collaboration (CKD-EPI) equation.

^†^Includes ex-smokers and current smokers.

We carried out a linear regression analysis to assess the relation between baseline characteristics and spirometric parameters. After controlling for age, body mass index (BMI), and extent of smoking, a multiple linear regression analysis showed that FVC was closely related to serum glucose, HbA1c, and 25 OH-vitamin D (Table 2). Also, FEV1was stronglyrelatedtoHbA1c, but other metabolic parameters showed no significant association (Table 3). We did not find a significant relationship between HbA1c and FEV_1_/FVC (data not shown).

**Table 2:** Multivariate linear regression for FVC (adjusted for age, body mass index and amount of smoking).

Variable	Univariate		Multivariate	
	Slope	P	Slope	P
Systolic blood pressure (mmHg)	-0.020358	0.0021	0.032747	0.5054
Waist circumference (cm)	-0.070421	0.0661		
Waist/height ratio	-8.700994	0.1403		
Conicity index (m^1½^·kg^-½^)	-4.937108	0.0807		
Hemoglobin (g/dL)	-0.095978	0.3872		
Glucose (mg/dL)	-0.052793	0.0028	0.148806	0.0113
HbA1c (%)	-3.001676	<0.0001	-6.972154	<0.0001
Triglyceride (mg/dL)	-0.003858	0.0268	-0.006127	0.1371
HDL-cholesterol (mg/dL)	0.045367	0.1181		
LDL-cholesterol (mg/dL)	-0.024528	0.0254	-0.027937	0.0764
25-vitamin D (ng/mL)	0.072487	0.0051	0.171532	0.0426
eGFR (mL·min^-1^·1.73 m^-2^)	-0.014914	0.3026		

**Table 3:** Multivariate linear regression for FEV_1_ (adjusted with age, body mass index and amount of smoking).

Variable	Univariate		Multivariate	
	Slope	P	Slope	P
Systolic blood pressure (mmHg)	-0.024381	0.0444	0.073470	0.1321
Waist circumference (cm)	-0.169096	<0.0001	0.037701	0.8697
Waist/height ratio	-11.254719	0.0843		
Conicity index (m^1½^·kg^-½^)	-10.34523	0.0007	-5.789909	0.7496
Hemoglobin (g/dL)	-0.175045	0.1380		
Glucose (mg/dL)	-0.057182	0.0030	0.084931	0.1139
HbA1c (%)	-3.249306	<0.0001	-5.591589	<0.0001
Triglyceride (mg/dL)	-0.005373	0.0017	-0.005252	0.2003
HDL-cholesterol (mg/dL)	0.075453	0.1001		
LDL-cholesterol (mg/dL)	-0.021962	0.0483	0.153414	0.0888
25-vitamin D (ng/mL)	0.080472	0.2646		
eGFR (mL·min^-1^·1.73 m^-2^)	-0.005255	0.7301		

### Restrictive spirometric pattern

**Fig 2:**
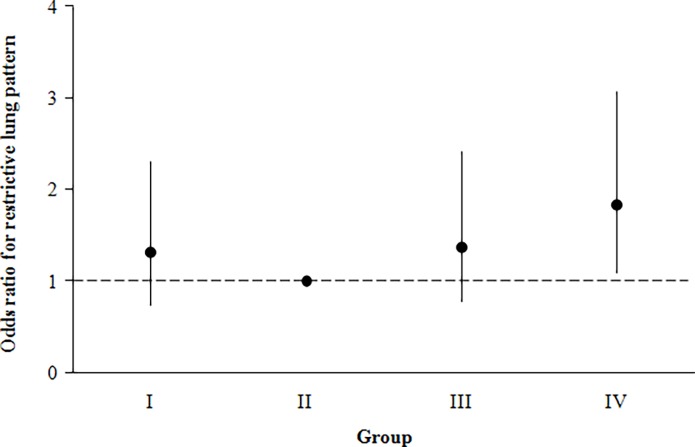
Adjusted multiple logistic regression analysis for the restrictive spirometric pattern. Adjusted for age, gender, body mass index, and amount of smoking.

Logistic regression analysis revealed that HbA1c was associated with obstructive and restrictive spirometric patterns. After adjustment for age, **gender**, BMI, WC, WHtR, C-index, triglyceride, LDL-cholesterol, and extent of smoking, logistic regression analysis demonstrated that HbA1c was a strong predictor of restrictive spirometric pattern (Table 4). However, we did not find a significant association between HbA1c and obstructive patterns (data not shown). Importantly, Fig. 2 shows that people with HbA1c > 5.7% appear to have an increased risk of developing the restrictive pattern: the adjusted OR of the restrictive spirometric pattern was 1.311 (95% *CI* = 0.745–2.308) for group I, 1.370 (95% *CI* = 0.776–2.417) for group III, and 1.837 (95% *CI* = 1.099–3.071) for group IV, using group II as reference.

**Table 4:** Logistic regression for restrictive spirometric pattern.

Variable	OR	95% *CI*
Age (year)	1.030	1.018–1.042
Female (*vs* male)	0.678	0.547–0.839
Systolic blood pressure (mmHg)	1.016	1.010–1.023
Body mass index (kg/m^2^)	1.146	1.101–1.193
Waist circumference (cm)	1.054	1.038–1.070
Waist/height ratio	999.9	20.55–999.9
Conicity index (m^1½^·kg^-½^)	259.9	48.76–999.9
Hemoglobin (g/dL)	1.108	1.027–1.195
Glucose (mg/dL)	1.029	1.017–1.042
HbA1c (%)	2.000	1.074–3.725
Triglyceride (mg/dL)	1.001	1.001–1.002
LDL-cholesterol (mg/dL)	1.010	1.003–1.017
25-vitamin D (ng/mL)	0.998	0.981–1.016
eGFR (mL·min^-1^·1.73 m^-2^)	0.995	0.985–1.004
Smoking amount (pack·years)	1.013	1.006–1.020
Adjusted for age, gender, body mass index, waist circumference, waist/height ratio, conicity index, triglyceride, LDL-cholesterol, and amount of smoking		
HbA1c (%)	3.772	1.234–11.530

OR, odds ratio

*CI*, confidence interval.

## Discussion

In the present study we showed that serum level of HbA1c was associated with decreased lung function parameters and could be of value in predicting restrictive spirometric patterns even below the accepted diagnostic threshold for DM. However, HbA1c was not independently associated with the obstructive lung function pattern in this study. These findings suggest that small increments of glycemic exposure, even below the threshold for diagnosis of DM, may be complicated by reduced lung volumes and airway flow limitation.

Although the pathophysiological mechanism underlying the association between glycemic exposure and abnormal lung function remains to be established, the major potential mechanisms are microangiopathy of the pulmonary vascular network and chronic low-grade tissue inflammation [17]. Previous studies have shown that dysglycemia affects lung connective tissue metabolism leading to thickening of the alveolar epithelial and endothelial basement membrane [18–21]. Furthermore, pulmonary microangiography is a characteristic manifestation of diabetes [22, 23]. Such findings results in reduced alveolar gas exchange in patients with type 1 and 2 DM. Chronic low-grade tissue inflammation may also contribute to a decline in lung function [24, 25]. A recent prospective study showed that elevated level of inflammatory markers was closely associated with an increased risk of future impairment of lung function [26]. Thus, the systemic inflammatory responses to metabolites in prediabetic or diabetic people may have a continuous negative effect on lung function.

There have been few studies of the association between HbA1c and mechanical abnormalities of the lung in patients without DM. In our linear regression analysis, HbA1c, a clinical indicator of glycemic exposure level over the previous 2–3 months, was inversely related to FVC and FEV_1_ in the general population. Previously, McKeever et al. showed that persons with elevated HbA1c had an associated decrease in FVC and FEV_1_ [27], and Lee et al. found that FVC, but not FEV_1_, decreased with increasing fasting glucose level [28]. Also, metabolic syndrome is closely associated with a restrictive spirometric pattern in the general population [29]. However, these studies included younger people and diabetic subjects, which may be a potential limitation to the finding whether long-term glycemic exposure without confounding effect of DM-related condition has a negative influence on spirometric values. To minimize factors affecting lung function, we excluded subjects under 40 years of age or with any clinical illness, and adjusted for confounding parameters; we then found that none of the components of metabolic syndrome——systolic BP, waist circumference, triglyceride, and high-density lipoprotein——were related to FVC and FEV_1_ except for blood glucose and HbA1c. Logistic regression analysis revealed that HbA1c was strongly associated with a restrictive pattern. Thus, this study showed that increased glycemic exposure, even within the normal blood glucose range, may have an adverse effect on lung function.

Our data also provide insight into the relationship between HbA1c level and impaired lung function in the non-diabetic population. Our logistic regression revealed that HbA1c was significantly associated with increased risk of a poor lung function, and that HbA1 > 5.7% may be an indicator of the restrictive lung pattern. HbA1c in the range 5.7–6.4% is considered as identifying individuals with prediabetes [30]. Because typical diabetic microvascular complications can occur in prediabetes through both glucose-related and glucose-independent mechanisms [9], our results provide clinical evidence that prediabetic ranged HbA1c may be important clue to find adverse effect of systemic inflammation on the lung.

There are several limitations to our study. First, the total lung capacity, a parameter for accurate diagnosis of restrictive lung disease, was not include as a pulmonary function test in KNHANES I-V [31]. Second, we did not assess the diffusing capacity of the lungs for carbon monoxide (DL_CO_). Decreased DL_CO_ has been documented as providing evidence of diabetic pulmonary microangiopathy [4]. Unfortunately, DL_CO_ also was not available in the KNHANES I-V. However, our results are consistent with other prospective or meta-analytic studies of diabetic patients that showed declines in both FVC and FEV_1_, but not in FEV_1_/FVC [32, 33]. Therefore, they may be sufficiently reliable to account for the relationship between HbA1c and poor lung function in the non-diabetic population. Third, our pulmonary function tests may have been influenced by a wide variety of factors. Because of study design, we could not adjust for many possible factors other than age, gender, BMI, and smoking level. Finally, because of the limitations of cross-sectional study, we did not examine long-term effects of glycemic exposure on the airway flow and lung parenchymal abnormalities.

The results of the present study suggest that relatively high HbA1c level in the healthy population is associated with decrements of FVC and FEV_1_ and is an important predictor of poor lung function, especially the restrictive pattern. However, a large population-based prospective clinical study is needed to identify the mechanisms involved and the clinical implications.
